# Seasonal Influenza Vaccination Uptake, Illness and Economic Burden, and Vaccine Information Exposure Among Young Adults in the San Francisco Bay Area

**DOI:** 10.3390/pharmacy14030087

**Published:** 2026-06-18

**Authors:** Taiwo Opeyemi Aremu, Carinne Brody, Shadi Doroudgar, Ikenna Chidozie Ezejiaku, Shahin Teimourtash

**Affiliations:** 1 Department of Clinical & Social Pharmaceutical Sciences, College of Pharmacy, Touro University, 1310 Club Dr., Vallejo, CA 94592, USA; sdoroudg@touro.edu (S.D.); iezejiaku@student.touro.edu (I.C.E.); steimourtash@student.touro.edu (S.T.); 2 Cardiac Rhythm Management (CRM), Medtronic, 8200 Coral Sea Street NE, Mounds View, MN 55112, USA; 3 Public Health Program, Touro University, 1310 Club Dr., Vallejo, CA 94592, USA; cbrody@touro.edu

**Keywords:** influenza, vaccination, young adults, flu vaccine, vaccine hesitancy, economic burden, community pharmacy, San Francisco Bay Area, cross-sectional survey

## Abstract

Background: Seasonal influenza prevention in young adults is influenced by access, trust, and vaccine information exposure, but local evidence linking vaccination uptake with illness and economic burden is limited. Methods: We conducted a non-probability, cross-sectional electronic survey of adults aged 18–49 years who lived, worked, or studied in the San Francisco Bay Area during the 2025 to 2026 influenza season. Measures included vaccination uptake, influenza-like illness, recovery, functional and economic burden, vaccination sites, and vaccine information exposure. Multivariable logistic regression examined factors associated with vaccination uptake; Kaplan–Meier and Cox models examined time to recovery. Results: Of 554 responses, 463 were included. Vaccination uptake was 86.2% (*n* = 399; 95% confidence interval [CI], 82.7–89.2%), likely reflecting a health-engaged convenience sample. Influenza-like illness was reported by 38.4%; median recovery time was 5 days, median missed work or school was 2 days, and median direct out-of-pocket cost was US$20. Prior season vaccination (adjusted odds ratio [aOR], 2.24; 95% CI, 1.15–4.34) and greater trust in Centers for Disease Control and Prevention (CDC) or public health agencies (aOR, 1.46; 95% CI, 1.05–2.02) were associated with vaccination. Pharmacies were the second most common vaccination site and preferred future site. Conclusions: Influenza prevention for young adults may benefit from pharmacy-inclusive, multichannel access paired with trusted communication. Findings should be interpreted in light of non-probability recruitment and likely overrepresentation of health-engaged respondents.

## 1. Introduction

Seasonal influenza remains a major cause of preventable morbidity, health care use, hospitalization, death, and economic loss in the United States. Between 2010 and 2025, influenza was estimated to cause 9.4 million to 51 million illnesses, 120,000 to 710,000 hospitalizations, and 6300 to 52,000 deaths annually in the United States [1]. The 2024 to 2025 influenza season was classified as a high severity season, with an estimated 51 million influenza illnesses, 23 million medical visits, 710,000 hospitalizations, and 45,000 deaths [2]. During the 2025 to 2026 season, CDC in-season surveillance estimated at least 31 million illnesses, 370,000 hospitalizations, and 23,000 deaths from influenza in the United States by early April 2026 [3].

Annual influenza vaccination remains the primary prevention strategy for reducing influenza-associated illness and complications. The Advisory Committee on Immunization Practices recommends routine annual influenza vaccination for all persons aged 6 months and older who do not have contraindications [4]. However, influenza vaccination coverage among adults remains below public health targets. During the 2024 to 2025 influenza season, overall vaccination coverage among U.S. adults aged 18 years and older was 41.9%, a decrease of 3.0 percentage points from the prior season [5]. Coverage was particularly low among adults aged 18 to 49 years, with CDC estimating coverage of approximately 31% during the 2024 to 2025 season [2]. This age group is important for prevention because adults aged 18 to 49 contributed an estimated 16% of influenza-related hospitalizations during the 2024 to 2025 season and were the age group in which vaccination prevented the largest share of influenza illnesses [2].

Recent vaccine effectiveness data further support the importance of vaccination, including among younger and working-age adults. Interim national estimates for the 2025 to 2026 season showed that influenza vaccination reduced the risk of influenza-associated outpatient visits by 22% to 34% among adults aged 18 years and older and reduced influenza-associated hospitalization by approximately 30% [6]. California-specific interim estimates from October 2025 through January 2026 also demonstrated vaccine benefit, with an overall vaccine effectiveness of 33% against laboratory-confirmed influenza and 34% among adults aged 18 to 49 years [7]. These California data are especially relevant to the present study because they reflect the same state context and the same influenza season in which this survey was conducted.

The burden of influenza is not limited to severe clinical outcomes. Influenza can also result in missed work or school, reduced usual activities, out-of-pocket expenses, and productivity losses. Earlier national estimates found that seasonal influenza generated approximately $10.4 billion in direct medical costs and $16.3 billion in projected lost earnings annually in the United States [8]. More recent estimates placed the average annual total economic burden of influenza at approximately $11.2 billion, including $3.2 billion in direct medical costs and $8.0 billion in indirect costs when lost productivity was included [9]. Among adults aged 18 to 64 years, the economic burden of influenza is largely indirect, reflecting missed work, productivity loss, and related functional disruption [10]. These outcomes are particularly relevant for young adults who may have fewer severe outcomes than older adults but may still experience meaningful economic and daily activity burden.

Influenza vaccination decisions are also shaped by perceived risk, access, trust, and exposure to vaccine-related information. In a national study of influenza-specific vaccine hesitancy among U.S. adults, 36.9% of adults reported hesitancy toward influenza vaccination, 18.6% reported concerns about vaccine side effects, and 35.6% reported that their health care provider was not their most trusted source of information about influenza vaccination [11]. These beliefs were associated with substantially lower vaccination coverage [11]. Major sources of influenza vaccine information include medical professionals, medical journals, newspapers, social media, family and friends, and promotional media. Prior research has shown that individuals who placed greater value on information from medical professionals and medical journals were more likely to perceive influenza vaccines as safe and effective and were subsequently more likely to receive influenza vaccination, whereas reliance on social media and informal sources was associated with lower perceived vaccine safety and efficacy and reduced vaccine uptake [12]. Because vaccine misinformation and conflicting health information may influence vaccination behavior, communication research has emphasized the importance of evaluating outcomes connected to actual vaccination uptake rather than only knowledge or attitudes [13].

Although national and state-level influenza data are available, less is known about how vaccination uptake, influenza-like illness, time to recovery, economic burden, and vaccine information exposure intersect among young adults in the San Francisco Bay Area. Young adults often move across work, school, transit, and social settings, and may receive vaccine information from diverse sources, including health professionals, public health agencies, traditional media, social media, family, and peers. Because young adults may obtain vaccination through several access points, including clinics, pharmacies, campuses, workplaces, and community sites, local evidence is needed to guide outreach strategies that reflect both where they receive vaccines and where they encounter vaccine-related information. Therefore, this study examined seasonal influenza vaccination uptake, influenza-like illness burden, time to recovery, economic burden, and vaccine information exposure among adults aged 18 to 49 years in the San Francisco Bay Area.

## 2. Materials and Methods

### 2.1. Study Design and Setting

We conducted a cross-sectional electronic survey among adults aged 18 to 49 years who lived, worked, or studied in the San Francisco Bay Area during the 2025 to 2026 influenza season. Data collection occurred from 9 January 2026 through 30 April 2026. The study included respondents from Alameda, Contra Costa, Marin, Napa, San Francisco, San Mateo, Santa Clara, Solano, and Sonoma counties. The survey was administered using Qualtrics and was designed to take approximately 10 min to complete.

This study examined seasonal influenza vaccination uptake, influenza-like illness, time to recovery, functional burden, economic burden, and vaccine information exposure among young adults. The economic component was conducted as an economic burden analysis rather than a cost-effectiveness analysis because respondents were not assigned to a vaccination intervention, vaccine effectiveness was not directly estimated, and incremental cost-effectiveness ratios were not calculated.

### 2.2. Participants and Recruitment

Eligible respondents were adults aged 18 to 49 years who reported living, working, or studying in one of the selected San Francisco Bay Area counties and who consented to participate. Respondents were excluded if they self-reported younger than 18 years, aged 50 years or older, did not live, work, or study in the Bay Area, did not consent, had inadequate survey completion, or had missing data on the primary outcome of seasonal influenza vaccination uptake. The upper age limit of 49 years was selected to focus on young and working-age adults whose vaccination coverage, exposure patterns, care-seeking behaviors, and information environments may differ from those of adults aged 50 years and older.

Respondents were recruited using a non-probability convenience sampling approach through Touro University California networks, partner campuses, community organizations, community settings, transit-adjacent locations, and online, social media, and messaging platforms, including Facebook, Instagram, Reddit, and WhatsApp. Recruitment materials included a brief description of the study, eligibility criteria, estimated survey duration, survey link, and QR code. To reduce duplicate participation, the Qualtrics “Prevent multiple submissions” setting was enabled; respondents detected as duplicates at the beginning of the survey were prevented from completing the survey more than once. The survey was anonymous and did not collect names, email addresses, or phone numbers within the survey dataset. Participants were given the option to enter a separate drawing for one of fifty US$20 electronic gift cards. Drawing entry information was collected through a separate claim form and was not stored with survey responses.

### 2.3. Sample Size

The target sample size was based on feasibility and precision for estimating the primary outcome, seasonal influenza vaccination uptake. A target of approximately 500 respondents was considered adequate to estimate a population proportion with a maximum 95% margin of error of approximately ±4.4 percentage points under the conservative assumption of *p* = 0.50. The final analytic sample of 463 respondents provided a maximum 95% margin of error of approximately ±4.6 percentage points for overall proportions. Analyses of influenza-like illness, time to recovery, and economic burden were conducted among smaller subgroups and were considered descriptive and exploratory.

### 2.4. Measures

The survey questionnaire included items on eligibility, county of residence and work or study, health access, seasonal influenza vaccination uptake, influenza-like illness, recovery and functional impact, direct and indirect costs, vaccine information exposure, trust in information sources, and demographics. The complete questionnaire, including response options and skip logic, is provided as Supplementary File S1.

The primary outcome was self-reported receipt of an influenza vaccine during the 2025 to 2026 influenza season. Responses were categorized as vaccinated, not vaccinated, or not sure. For the primary analysis, vaccination uptake was coded as vaccinated versus not vaccinated or not sure.

Demographic and health access variables included age group, gender, race, Hispanic or Latino ethnicity, Bay Area county, education, household income, student status, employment status, work or study setting, health insurance, and preferred vaccination location. Health status was assessed using self-reported clinician-diagnosed conditions, including asthma or chronic lung disease, diabetes, heart or kidney disease, weakened immune system, and pregnancy during the influenza season. A binary variable was created for any chronic condition.

Health insurance was self-reported using the original survey categories of private insurance, Medi-Cal, Medicare, student health plan, uninsured, and prefer not to say. Original categories were retained for descriptive analyses. Because Medicare may be reported by adults younger than 65 years under specific eligibility pathways but may also be confused with Medi-Cal in a California sample, the primary adjusted analysis used a collapsed insurance variable with three categories: private or student health plan, public insurance, and uninsured or unknown. Public insurance included respondents who reported Medi-Cal or Medicare. Insurance findings were interpreted as indicators of self-reported health coverage and access rather than definitive payer classification.

Influenza-like illness was defined for respondents as fever with cough or sore throat. Among respondents who reported influenza-like illness, the survey assessed the number of illness episodes, influenza testing, COVID-19 testing, care seeking, antiviral prescription, hospitalization, care or help received from others, paid sick leave, missed work or school, reduced usual activities, missed paid work hours, missed unpaid work or caregiving hours, and time to recovery. The antiviral prescription item referred to prescription treatment for influenza, such as oseltamivir (Tamiflu) or another influenza antiviral medication. Time to recovery was defined as the number of days from symptom onset until the respondent felt recovered enough to return to usual activities. Respondents who had not fully recovered at the time of survey completion were treated as censored observations when sufficient timing information was available.

Economic burden was assessed among respondents who reported influenza-like illness. Direct costs included self-reported out-of-pocket costs for medical visits and copays, medications, transportation or delivery related to care or supplies, and other illness-related expenses. Total direct out-of-pocket cost was calculated as the sum of these direct cost components. Indirect burden included missed paid work hours, missed unpaid work or caregiving hours, missed work or school days, reduced usual activities, care received from others, and paid sick leave status.

Vaccine information and trust assessed exposure to negative flu vaccine claims, exposure to conflicting vaccine information, perceived credibility of negative claims, likelihood of fact-checking or looking for official sources, confidence in identifying accurate flu vaccine information, difficulty knowing which online health information to trust, and trust in health professionals, the CDC or other public health agencies, social media influencers, family and friends, and traditional news media. These items were developed for this study to align with the survey objectives and were not adapted from a single validated multi-item instrument. Because the items captured distinct constructs rather than one underlying scale, no composite score was created and internal consistency was not assessed.

### 2.5. Statistical Analysis

Descriptive statistics were used to summarize respondent characteristics, vaccination uptake, influenza-like illness burden, time to recovery, economic burden, and vaccine information variables. Categorical variables were reported as frequencies and percentages. Continuous variables were reported as means and standard deviations or medians and interquartile ranges, depending on distribution.

Influenza vaccination uptake was estimated as the proportion of respondents who reported vaccination during the 2025 to 2026 influenza season, with 95 percent confidence intervals. Bivariable associations between vaccination uptake and categorical variables were examined using chi-square tests or Fisher’s exact tests, as appropriate. Continuous and ordinal variables were compared using Wilcoxon rank-sum tests or Kruskal–Wallis tests. Vaccine information and trust items were analyzed individually. Likert-style responses were coded from 1 to 5, with higher scores reflecting greater credibility, fact-checking likelihood, confidence, difficulty trusting online information, or trust, depending on the item. For descriptive and bivariable analyses, these variables were summarized using means and standard deviations and compared by vaccination status using Wilcoxon rank-sum or Kruskal–Wallis tests.

A parsimonious multivariable logistic regression model was used to examine factors associated with influenza vaccination uptake. The outcome was current-season influenza vaccination, modeled as vaccinated versus not vaccinated or not sure. Candidate predictors were selected based on conceptual relevance and cell size adequacy rather than automated stepwise selection. The primary adjusted model included age group, collapsed gender, collapsed race, collapsed insurance category, chronic condition status, prior season influenza vaccination, frequency of exposure to negative vaccine claims, perceived credibility of negative claims, fact-checking score, confidence in identifying accurate vaccine information, trust in health professionals, and trust in the CDC or other public health agencies. Selected 1-to-5 vaccine information and trust variables were modeled as continuous exploratory predictors to limit model complexity, and estimates were interpreted as the change in odds of vaccination per 1-point increase. Respondents with missing covariate data for variables included in the adjusted model were excluded using complete-case analysis; no imputation was performed for the primary regression model. Adjusted odds ratios and 95% confidence intervals were reported. Sensitivity analyses excluded respondents who selected “not sure” for vaccination status and examined the influence of insurance coding, including analyses using original insurance categories and analyses excluding respondents who reported Medicare.

Among respondents with influenza-like illness, time to recovery was summarized using medians and interquartile ranges. Kaplan–Meier methods were used to estimate time to recovery, with recovery to usual activities treated as the event [14]. Log-rank tests compared time to recovery by vaccination status. An exploratory Cox proportional hazards model examined factors associated with time to recovery [15]. The proportional hazards assumption for the exploratory Cox model was assessed descriptively using graphical diagnostics where data permitted; interpretation was limited by the small time-to-recovery analytic sample and the very small number of censored observations. A hazard ratio greater than 1 indicated faster recovery, while a hazard ratio less than 1 indicated slower recovery. These analyses were interpreted cautiously because the study was cross-sectional, timing measures were self-reported, and the number of unvaccinated respondents with influenza-like illness was small.

Economic burden analyses were conducted among respondents reporting influenza-like illness. Cost and missed-time variables were inspected for nonnumeric entries, negative values, and extreme responses. Nonnumeric, negative, or otherwise invalid values were treated as missing for the relevant analysis; zero values were retained as true zero costs or hours. Extreme values were reviewed and retained unless they were clearly implausible based on the survey item and response context. No imputation was performed for cost or missed-time variables. Direct and indirect burden variables were summarized using medians and interquartile ranges, with means and standard deviations reported for completeness. Medians were emphasized because cost and missed work variables were right-skewed. Economic burden measures were compared by vaccination status using Wilcoxon rank-sum or Kruskal–Wallis tests. These analyses were interpreted as descriptive and exploratory because the study was not designed to estimate the causal effect of vaccination on costs.

The level of significance (alpha) was set to 0.05. All analyses were conducted using SAS 9.4 OnDemand for Academics (SAS Institute Inc., Cary, NC, USA).

### 2.6. Ethical Considerations

The study protocol was approved via expedited review by the Touro University California Institutional Review Board under IRB application #P-0825 on 7 November 2025. The study used an anonymous survey design. Participation was voluntary. Incentive claim information was collected separately from survey responses and was not linked to the survey dataset. Respondents provided informed consent before completing the survey.

## 3. Results

### 3.1. Respondent Characteristics

**Figure 1:**
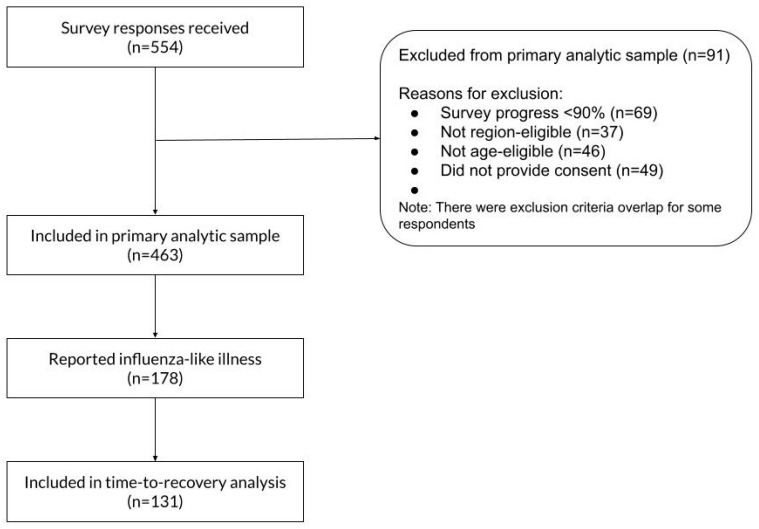
Survey response disposition and analytic sample.

A total of 554 survey responses were received. Of these, 485 had survey progress of at least 90%, 517 met regional eligibility criteria, 508 met age eligibility criteria, and 505 provided consent. After applying completion, eligibility, consent, and primary outcome criteria, the final analytic sample included 463 respondents. Among these, 178 respondents reported influenza-like illness and 131 respondents were included in the time-to-recovery analysis (Figure 1).

Most respondents were aged 25 to 34 years (*n* = 260, 56.2%). Approximately half identified as women (*n* = 235, 50.8%). By race, nearly half identified as White (*n* = 233, 50.3%), followed by Black or African American (*n* = 100, 21.6%) and Asian (*n* = 64, 13.8%). A total of 75 respondents (16.2%) identified as Hispanic or Latino. Nearly half of the respondents were students (*n* = 229, 49.5%), and 277 respondents (59.8%) were employed. A total of 119 respondents (25.7%) reported at least one clinician-diagnosed chronic condition. Participant characteristics are summarized in Table 1.

Self-reported original insurance categories were retained descriptively. Private insurance and Medicare were each reported by 160 respondents (34.6% each), followed by Medi-Cal (*n* = 83, 17.9%), student health plan (*n* = 49, 10.6%), prefer not to say (*n* = 8, 1.7%), and uninsured (*n* = 3, 0.7%). For adjusted analyses, Medi-Cal and Medicare were grouped as public insurance.

### 3.2. Influenza Vaccination Uptake

Overall, 399 of 463 respondents reported receiving an influenza vaccine during the 2025 to 2026 season, corresponding to a vaccination uptake of 86.2% (exact 95% CI: 82.7–89.2%). Prior season influenza vaccination was reported by 315 respondents (68.0%). Among vaccinated respondents, October 2025 was the most common month of vaccination. The most common vaccination site was a doctor’s office or clinic, followed by a pharmacy and workplace or campus event. Pharmacy was also the second most preferred future vaccination site after a doctor’s office or clinic. Vaccination characteristics are shown in Table 2.

In bivariable analyses, vaccination uptake differed by collapsed insurance category (*p* = 0.0001), prior season influenza vaccination (*p* = 0.0059), student status (*p* = 0.0007), employment status (*p* = 0.0005), and frequency of exposure to negative flu vaccine claims (*p* = 0.0331). Vaccination uptake did not differ significantly by age group, collapsed gender, ethnicity, chronic condition status, or work or study setting.

### 3.3. Multivariable Factors Associated with Influenza Vaccination Uptake

The primary multivariable logistic regression model included 430 respondents after excluding observations with missing covariates. The model converged and showed overall evidence of association between the selected predictors and vaccination uptake. In the adjusted model, prior season influenza vaccination and greater trust in the CDC or other public health agencies were significantly associated with higher odds of current season vaccination. Any clinician-diagnosed chronic condition was associated with lower odds of vaccination, but the association was borderline significant. Adjusted results are shown in Table 3.

Sensitivity analyses supported the main findings. When respondents who selected “not sure” for vaccination status were excluded, prior season vaccination remained associated with current vaccination (OR, 2.14; 95% CI: 1.09–4.20; *p* = 0.027), as did trust in the CDC or other public health agencies (OR, 1.42; 95% CI: 1.01–1.98; *p* = 0.042). In a sensitivity analysis excluding respondents who reported Medicare, trust in public health agencies remained associated with vaccination uptake, while exposure to negative flu vaccine claims and fact-checking score also became statistically significant.

### 3.4. Influenza-like Illness Burden and Functional Impact

Overall, 178 respondents (38.4%) reported influenza-like illness during the season. Among those reporting influenza-like illness, most reported one episode, while approximately one-third reported two or more episodes. Testing, care seeking, and antiviral prescription were common among respondents reporting influenza-like illness. Functional burden included time to recovery, missed work or school, reduced usual activities, and need for care or help from another person (Table 4).

Among respondents with influenza-like illness and available recovery data, the median time to recovery was 5 days (IQR, 3–7 days). Median missed work or school was 2 days (IQR, 0–5 days), and median reduced usual activities was 3 days (IQR, 0–5 days). Among respondents who received help from another person, the median amount of care help received was 10 h (IQR, 5–32 h).

### 3.5. Time-to-Recovery Analysis

Kaplan–Meier time-to-recovery analysis included 131 respondents, of whom 130 recovered and 1 was censored. Median time to recovery was 5 days among respondents who were not vaccinated or not sure (95% CI, 3–6 days) and 5 days among vaccinated respondents (95% CI: 4–5 days). The log-rank test showed no significant difference in time to recovery by vaccination status (*p* = 0.694).

In the exploratory Cox proportional hazards model, vaccination status was not significantly associated with time to recovery (HR, 0.78; 95% CI: 0.44–1.40; *p* = 0.408). Care seeking was associated with slower recovery (HR, 0.47; *p* = 0.008), likely reflecting greater illness severity among respondents who sought care. Chronic condition status was directionally associated with slower recovery but did not reach statistical significance (HR, 0.68; *p* = 0.064).

### 3.6. Economic Burden and Missed-Time Outcomes

Among respondents reporting influenza-like illness, direct out-of-pocket costs were right-skewed. The median total direct out-of-pocket cost was US$20 (IQR, US$0–US$140). Median costs for individual direct cost components were US$0, although upper quartiles indicated that some respondents incurred medical visits, medication, transportation, delivery, or other illness-related costs (Table 5). Means and standard deviations are also reported in Table 5 for completeness but were not emphasized because a small number of high-cost reports increased the mean.

When economic burden was compared by vaccination status among respondents with influenza-like illness, there were no statistically significant differences in total direct out-of-pocket costs, missed paid work hours, missed unpaid work or caregiving hours, missed work or school days, or reduced activity days (Table A1).

### 3.7. Vaccine Information Exposure, Credibility, Trust, and Fact Checking

Exposure to negative flu vaccine claims was common. Overall, 416 respondents (89.9%) reported exposure to negative flu vaccine claims at least rarely, while 41 respondents (8.9%) reported never being exposed. More than half of respondents (*n* = 251, 54.2%) reported exposure to conflicting vaccine information. In scenario-based items, 307 respondents (66.3%) reported that they would look up additional information, 103 (22.3%) reported that they would believe the information, 69 (14.9%) reported that they would share it, and 94 (20.3%) reported that they would ignore it. Only 29 respondents (6.3%) reported that online information had changed their opinion about flu vaccines. Vaccine information and trust scores by vaccination status are shown in Table 6.

Vaccinated respondents reported higher confidence in identifying accurate flu vaccine information than respondents who were not vaccinated or not sure (mean, 3.64 vs 3.29; *p* = 0.012). Vaccinated respondents also reported greater trust in the CDC or other public health agencies (mean, 3.74 vs 3.32; *p* = 0.002) and greater trust in traditional news media (mean, 3.11 vs 2.81; *p* = 0.049). Trust in health professionals and social media influencers was directionally higher among vaccinated respondents but did not reach statistical significance. Credibility of negative flu vaccine claims, fact-checking likelihood, difficulty knowing which online information to trust, and trust in family and friends did not differ significantly by vaccination status.

## 4. Discussion

This cross-sectional survey of adults aged 18 to 49 years in the San Francisco Bay Area found high self-reported seasonal influenza vaccination uptake, with 86.2% of respondents reporting vaccination during the 2025 to 2026 season. Despite this high uptake, influenza-like illness was common, reported by 178 respondents (38.4%), and was associated with functional and economic burden, including a median recovery time of 5 days, missed work or school, reduced usual activities, and out-of-pocket costs. In adjusted analyses, prior season influenza vaccination and greater trust in the CDC or other public health agencies were associated with higher odds of current season vaccination, while vaccination status was not significantly associated with time to recovery or economic burden among respondents reporting influenza-like illness. Because this was a non-probability convenience sample, the high uptake should be interpreted as describing a reachable and relatively health-engaged local sample rather than all Bay Area young adults.

Vaccination uptake in this study was higher than national estimates and public health targets. CDC estimates for the 2024 to 2025 season showed adult influenza vaccination coverage of 41.9% overall, with lower coverage among adults aged 18 to 49 years [2,5], and the observed uptake also exceeded the Healthy People 2030 target of 70% for annual influenza vaccination [16]. This contrast likely reflects recruitment through university, community, and online networks that reached more health-engaged respondents. Nevertheless, national data underscore the relevance of young adults for influenza prevention because adults aged 18 to 49 years had low national vaccination coverage but accounted for the largest share of influenza illnesses prevented by vaccination during the 2024 to 2025 season [2].

The high uptake in this sample also differs from prior studies of university students and young adults. Bednarczyk and colleagues reported seasonal influenza vaccine uptake of 28% among students at a large public university, with perceived low need, safety concerns, and other barriers contributing to non-vaccination [17]. Nearly half of the respondents in the present sample were students, but uptake was much higher than in that university-based study. This difference may reflect the Bay Area context, the timing of data collection, recruitment through health-related and community networks, or greater health engagement among respondents who chose to participate.

The association between prior season vaccination and current season vaccination is consistent with evidence that influenza vaccination behavior tends to be stable across adjacent seasons. Walsh and colleagues found that influenza vaccination behavior can remain consistent over time, suggesting that vaccination may become a repeated seasonal habit rather than a new decision each year [18]. A systematic review of influenza vaccine hesitancy also identified prior vaccination behavior, confidence, complacency, and barriers to access as important contributors to influenza vaccination intention and behavior [19]. This finding suggests that recurring campus clinics, workplace vaccination events, pharmacy reminder systems, and clinician prompts may help reinforce repeat vaccination behavior, although the present study was not designed to test these interventions.

The trust and information findings align with prior literature on vaccine confidence, information sources, and hesitancy. Greater trust in the CDC or other public health agencies was associated with higher odds of current season vaccination in this study. Lohmann and Albarracín similarly found that trust in the CDC as a vaccine information source was related to vaccination uptake, particularly when broader conditions supported access to vaccination [20]. Influenza-specific hesitancy research has also shown that concerns about side effects, uncertainty about vaccine benefits, and lack of trust in information sources are associated with lower vaccination coverage [11]. Hwang further showed that health information sources may influence influenza vaccination through perceived vaccine efficacy and safety, reinforcing the importance of both trusted channels and message content [12]. In the present study, exposure to negative vaccine claims was common, but perceived credibility of those claims was not independently associated with vaccination uptake. This suggests that exposure alone may be less consequential than how respondents evaluate what they encounter, whether they trust public health sources, and how confident they feel in identifying accurate flu vaccine information.

The vaccine information findings are important because nearly 90% of respondents reported at least some exposure to negative flu vaccine claims, and more than half reported exposure to conflicting vaccine information. Prior reviews have emphasized that communication interventions addressing vaccine misinformation should be evaluated using behavioral outcomes, including vaccination uptake, rather than knowledge or attitudes alone [13]. The present findings support that view. Respondents were often exposed to negative or conflicting information, but vaccination was more closely tied to trust in public health agencies and confidence in identifying accurate information. The vaccine information and trust items were developed for this study and were analyzed as individual indicators rather than as a single psychometric scale. Therefore, these findings should be interpreted as item-specific measures of vaccine information exposure, confidence, and trust. The illness and economic burden findings are consistent with prior evidence that influenza creates meaningful functional and productivity-related burden among working-age adults [8,9,10]. In this study, respondents with influenza-like illness reported a median recovery time of 5 days, median missed work or school of 2 days, median reduced usual activities of 3 days, and median total direct out-of-pocket costs of US$20. Because cost variables were right-skewed, these findings was interpreted primarily using medians and interquartile ranges rather than means. Although the median total direct out-of-pocket cost was modest, the upper quartile showed that some respondents incurred meaningful illness-related costs. These findings support the interpretation that influenza-like illness was associated with functional and economic disruption among young adults, while avoiding overinterpretation of mean costs influenced by a small number of high-cost reports.

The absence of a significant association between vaccination status and time to recovery or economic burden should not be interpreted as evidence against vaccine benefit. This study relied on self-reported influenza-like illness and was not designed to estimate vaccine effectiveness. By contrast, interim estimates from the 2025 to 2026 season demonstrated that influenza vaccination reduced influenza-associated outpatient visits and hospitalizations among adults nationally [6]. California-specific interim estimates from the same season also showed vaccine benefit against laboratory-confirmed influenza [7]. Those findings require laboratory-confirmed outcomes and study designs intended to measure protection, whereas the present study was designed to describe self-reported uptake, illness experience, recovery, cost, and information exposure. Because of the cross-sectional design, associations between vaccination status and influenza-like illness, recovery time, and economic burden should not be interpreted as causal effects.

The vaccination location findings provide a natural link to pharmacy practice because pharmacies appeared in both reported vaccination locations and preferred future vaccination sites. This pattern is consistent with national data from the 2023 to 2024 respiratory virus season showing that pharmacies and drug stores were major settings for adult influenza vaccination, along with doctor’s offices and other clinical settings [21]. It is also consistent with pharmacy literature showing that community pharmacies can serve as feasible adult vaccination sites and that pharmacist involvement as immunizers, educators, or facilitators can increase vaccination coverage [22,23]. For young adults who move across school, work, transit, and community settings, these findings support a coordinated access model in which clinics, pharmacies, campuses, workplaces, and community sites complement one another.

These findings suggest several practical considerations for local influenza prevention efforts among young adults. Vaccination programs may consider treating annual influenza vaccination as a recurring seasonal behavior rather than a one-time decision. Because prior season vaccination was associated with current season uptake, recurring reminder systems, annual vaccine events, and routine prompts in campuses, workplaces, pharmacies, and clinics may be useful areas for implementation or evaluation.

Communication strategies may also benefit from strengthening trust in credible sources, not only correcting misinformation after it has already circulated. Respondents were frequently exposed to negative or conflicting flu vaccine information, yet trust in public health agencies was associated with vaccination uptake. Messages from public health agencies, clinicians, pharmacists, and community partners should therefore be clear, consistent, and practical, emphasizing where to get vaccinated, what to expect, and why vaccination remains useful for young adults.

Prebunking may be a useful complement to traditional vaccine communication. Rather than responding only after misinformation spreads, prebunking aims to build resistance by warning people about common misinformation tactics before full exposure occurs. Experimental work suggests that inoculation-style interventions can improve resistance to misleading information by helping people recognize manipulative techniques such as emotional appeals, false expertise, and misleading argument structures [24,25,26]. Broader research on misinformation also emphasizes that false information operates within complex information ecosystems and may require approaches that build reasoning, source evaluation, and resilience rather than correction alone [27,28]. Applied to influenza prevention, prebunking could be used in brief pharmacy, clinic, campus, or social media messages that help young adults recognize common flu vaccine myths before they encounter them. However, such approaches should be tested in influenza-specific settings and evaluated using behavioral outcomes, such as vaccination uptake, not only attitudes or knowledge [13,29].

Vaccine access should remain flexible and multichannel. Doctor’s offices, clinics, and pharmacies were important vaccination settings, but young adults may also benefit from options in campus, workplace, transit-adjacent, and community settings. Pairing convenient access with trusted communication may be especially useful for young adults who balance school, work, commuting, and changing schedules. For pharmacy practice, the findings support consideration of community pharmacies not only as vaccine administration sites but also as settings where pharmacists can reinforce public health messages, address safety and effectiveness questions, direct patients to reliable sources, and support annual vaccination routines.

Although the numerical vaccination uptake estimate should be interpreted as specific to this non-probability Bay Area sample, several practice implications may be transferable to other settings. The importance of repeat seasonal vaccination behavior, trust in credible public health and health professional sources, multichannel vaccine access, and the ability to evaluate vaccine information are not unique to the Bay Area or the United States. These themes may be relevant to other urban, university-connected, or community-based settings where young adults move across school, work, community, pharmacy, and digital environments. However, their application will depend on local vaccination policy, pharmacy scope of practice, health system financing, vaccine availability, and public trust in health institutions.

### 4.1. Strengths and Limitations

This study has several strengths. It provides local evidence on influenza vaccination uptake, influenza-like illness, recovery burden, economic burden, and vaccine information exposure among young adults in the San Francisco Bay Area, a population that is mobile across work, school, transit, and social settings but is less often the focus of influenza burden studies. The study also captured multiple dimensions of influenza prevention and burden in the same sample, including vaccination behavior, time to recovery, missed work or school, direct and indirect costs, trust in information sources, and exposure to negative or conflicting flu vaccine claims. In addition, the analysis used methods appropriate to the data structure, including nonparametric summaries for skewed cost variables, Kaplan–Meier methods for time to recovery, and sensitivity analyses for vaccination status and insurance coding.

This study also has limitations. The cross-sectional design limits causal inference. In addition, the non-probability convenience sampling approach may have introduced selection bias by disproportionately reaching respondents who were more health-engaged, health-literate, vaccine-favorable, or digitally connected than the broader population of adults aged 18 to 49 years in the San Francisco Bay Area. This potential selection bias may partly explain the high vaccination uptake and limits the generalizability of the findings to all young and working-age adults in the region. All key measures were self-reported, including vaccination status, influenza-like illness, time to recovery, care seeking, and costs. Influenza-like illness was symptom-based and not consistently laboratory confirmed, so some reported episodes may have reflected other respiratory infections. In addition, the vaccine information and trust items were developed for this study and were not intended to function as a single psychometric scale; therefore, findings from these items should be interpreted as item-specific indicators of vaccine information exposure, confidence, and trust rather than as a composite measure. Some variables also required cautious interpretation, including self-reported insurance type, where Medicare may have been confused with Medi-Cal, and hospitalization, which may have been interpreted differently by respondents. Finally, economic analyses were descriptive and should be interpreted as economic burden rather than cost-effectiveness, because the study did not estimate vaccine effectiveness or causal cost differences.

### 4.2. Future Research

Future research should use longitudinal or prospective designs to better establish the timing between vaccination, influenza-like illness, recovery, and economic burden. Studies with probability-based or targeted sampling could improve representativeness and oversample unvaccinated young adults to better understand barriers to uptake. Future work could also validate influenza-like illness using laboratory testing or medical records where feasible. Studies that test clinic-, pharmacy-, campus-, and workplace-partnered outreach models could help determine which combinations of access and communication strategies are most effective for young adults in different health system contexts. Research that evaluates prebunking and psychological inoculation interventions in the context of influenza vaccination could also help determine whether building capacity to identify and resist misinformation supports vaccine confidence and uptake among young adults in local and community settings. Finally, the cost and recovery estimates from this study could help inform formal economic models of targeted influenza vaccination outreach, but such models would need external estimates of vaccine effectiveness, vaccine delivery costs, and avoided illness.

## 5. Conclusions

This study suggests that influenza prevention among young adults should extend beyond vaccine availability alone. In this health-engaged, non-probability Bay Area sample, high reported vaccination uptake occurred alongside illness-related disruption, out-of-pocket costs, missed activities, and frequent exposure to negative or conflicting vaccine information. The findings add local evidence that young adults may experience meaningful functional and economic burden from influenza-like illness, while also highlighting the role of trust in public health sources and confidence in identifying accurate vaccine information. Local prevention efforts may be strengthened by pairing convenient vaccination access through clinics, pharmacies, campuses, workplaces, and community settings with credible communication from trusted health professionals and public health agencies. Strategies that build capacity to evaluate and resist misinformation including prebunking or psychological inoculation approaches that proactively expose individuals to common misinformation tactics before full exposure occurs, may complement traditional communication efforts and support vaccine information confidence among young adults.

## Figures and Tables

**Table 1:** Participants characteristics of the analytic sample.

Characteristic	Category	*n* (%)
Age group (years)	18–24	111 (24.0)
	25–34	260 (56.2)
	35–44	72 (15.6)
	45–49	20 (4.3)
Gender	Woman	235 (50.8)
	Man	216 (46.7)
	Another, not disclosed, or missing	12 (2.6)
Race	White	233 (50.3)
	Black or African American	100 (21.6)
	Asian	64 (13.8)
	Other or multiple races	51 (11.0)
	Not disclosed or missing	15 (3.2)
	Hispanic or Latino	75 (16.2)
County of residence	San Francisco	166 (35.9)
	Alameda	68 (14.7)
	Solano	58 (12.5)
	Contra Costa	54 (11.7)
	Santa Clara	36 (7.8)
	Napa	23 (5.0)
	Marin	19 (4.1)
	Sonoma	19 (4.1)
	San Mateo	13 (2.8)
	Outside the Bay Area, prefer not to say, or missing	7 (1.5)
Insurance	Public insurance	243 (52.5)
	Private or student health plan	209 (45.1)
	Uninsured, unknown, or prefer not to say	11 (2.4)
Work or study setting	In person	222 (48.0)
	Hybrid	169 (36.5)
	Remote	71 (15.3)
	Not applicable	1 (0.2)

**Table 2:** Influenza vaccination uptake and vaccination characteristics.

Measure	Category	*n* (%)	Denominator
Current season influenza vaccination	Vaccinated	399 (86.2)	463
	Not vaccinated or not sure	64 (13.8)	
Month vaccinated among vaccinated respondents	August 2025	32 (8.0)	399
	September 2025	60 (15.0)	
	October 2025	141 (35.3)	
	November 2025	71 (17.8)	
	December 2025	47 (11.8)	
	January 2026	46 (11.5)	
	Not sure	2 (0.5)	
Vaccination location among vaccinated respondents	Doctor’s office or clinic	221 (55.4)	399
	Pharmacy	97 (24.3)	
	Workplace or campus event	60 (15.0)	
	Community site	20 (5.0)	
	Other	1 (0.3)	
Preferred future vaccination site	Doctor’s office or clinic	274 (59.2)	463
	Pharmacy	112 (24.2)	
	Workplace or campus event	37 (8.0)	
	Community site	19 (4.1)	
	I do not plan to get a vaccine	7 (1.5)	
	Other, place of worship, or missing	14 (3.0)	

**Table 3:** Adjusted factors associated with current season influenza vaccination.

Variable	aOR	95% CI	*p*-Value
Age 25–34 years [Ref: 18–24 years]	1.60	0.76–3.40	0.218
Age 35–44 years [Ref: 18–24 years]	1.00	0.37–2.70	0.997
Age 45–49 years [Ref: 18–24 years]	0.45	0.12–1.62	0.220
Woman [Ref: Man]	1.48	0.78–2.84	0.233
Asian [Ref: White]	0.70	0.28–1.76	0.447
Black or African American [Ref: White]	0.60	0.28–1.26	0.175
Other or multiple races [Ref: White]	1.77	0.53–5.91	0.351
Public insurance [Ref: private or student plan]	1.49	0.76–2.93	0.242
Uninsured, unknown, or prefer not to say [Ref: private or student plan]	0.29	0.05–1.72	0.172
Any clinician-diagnosed chronic condition	0.51	0.26–1.00	0.051
Prior season influenza vaccination	2.24	1.15–4.34	0.017
Exposure to negative flu vaccine claims, per category increase	1.37	0.96–1.96	0.083
Credibility of negative flu vaccine claims, per 1-point increase	1.13	0.84–1.52	0.411
Fact-checking likelihood, per 1-point increase	1.18	0.87–1.59	0.297
Confidence identifying accurate flu vaccine information, per 1-point increase	1.33	0.95–1.87	0.102
Trust in health professionals, per 1-point increase	1.08	0.75–1.55	0.673
Trust in CDC or public health agencies, per 1-point increase	1.46	1.05–2.02	0.024

Note: aOR = adjusted odds ratio; CI = confidence interval. The model included 430 complete cases; 33 respondents were excluded because of missing covariate data. No imputation was performed. The outcome was vaccinated versus not vaccinated or not sure. Reference categories were 18 to 24 years for age, man for gender, White for race, and private or student health plan for insurance. Insurance, race, and gender were collapsed for the primary adjusted model.

**Table 4:** Influenza-like illness and functional burden.

Outcome	Category	*n* (%) or Median (IQR)
Influenza-like illness (N = 463)	Yes	178 (38.4)
	No or not sure	285 (61.6)
Number of episodes among respondents with influenza-like illness (N = 178)	One episode	106 (59.6)
	Two episodes	54 (30.3)
	Three or more episodes	15 (8.4)
	Missing	3 (1.7)
Clinical and care-seeking indicators among respondents with influenza-like illness (N = 178)	Any influenza test	145 (81.5)
	Positive influenza test	114 (64.0)
	Any COVID test	150 (84.3)
	Sought care	148 (83.1)
	Antiviral prescription	120 (67.4)
	Self-reported hospitalization	66 (37.1)
	Received care or help from another person	106 (59.6)
Functional burden among respondents with available data	Time to recovery (days), *n* = 130	5 (3–7)
	Missed work or school (days), *n* = 135	2 (0–5)
	Reduced usual activities (days), *n* = 122	3 (0–5)
	Care help received (hours), *n* = 57	10 (5–32)
	Missed paid work (hours), *n* = 118	12 (0–25)
	Missed unpaid work or caregiving (hours), *n* = 106	0 (0–10)

Note: Hospitalization was self-reported and was not clinically verified. Functional burden summaries are presented among respondents with available data.

**Table 5:** Direct and indirect economic burden among respondents with influenza-like illness.

Economic or Functional Burden Measure	*n*	Median (IQR)	Mean (SD)
Medical visit or copay cost, US$	177	0 (0–50)	191.73 (675.29)
Medication cost, US$	170	0 (0–39)	98.83 (305.86)
Transportation or delivery cost, US$	170	0 (0–20)	68.38 (205.86)
Other out-of-pocket cost, US$	170	0 (0–10)	46.59 (195.76)
Total direct out-of-pocket cost, US$	177	20 (0–140)	397.08 (1086.90)
Missed paid work, hours	118	12 (0–25)	20.94 (32.27)
Missed unpaid work or caregiving, hours	106	0 (0–10)	9.94 (24.75)
Missed work or school, days	135	2 (0–5)	8.35 (24.27)
Reduced usual activities, days	122	3 (0–5)	5.22 (10.61)

Note: Medians and interquartile ranges are the primary summaries because cost and missed work variables were right-skewed. Means and standard deviations are shown for completeness.

**Table 6:** Vaccine information, credibility, trust, and fact-checking scores by vaccination status.

Variable	Overall Mean (SD)	Not Vaccinated or Not Sure Mean (SD)	Vaccinated Mean (SD)	*p*-Value
Credibility of negative flu vaccine claims	2.36 (1.27)	2.27 (1.13)	2.37 (1.29)	0.666
Fact-checking likelihood	4.01 (1.12)	3.98 (1.12)	4.02 (1.12)	0.675
Confidence identifying accurate flu vaccine information	3.59 (0.95)	3.29 (1.02)	3.64 (0.93)	0.012
Difficulty knowing which online health information to trust	3.05 (1.15)	3.19 (1.15)	3.03 (1.15)	0.290
Trust in health professionals	3.99 (0.95)	3.79 (1.02)	4.02 (0.94)	0.076
Trust in CDC or other public health agencies	3.68 (0.99)	3.32 (1.04)	3.74 (0.98)	0.002
Trust in social media influencers	2.77 (1.30)	2.49 (1.16)	2.81 (1.32)	0.079
Trust in family and friends	3.22 (1.15)	3.21 (1.06)	3.23 (1.17)	0.990
Trust in traditional news media	3.07 (1.18)	2.81 (1.16)	3.11 (1.18)	0.049

Note: Scores were measured on 1 to 5 scales, with higher scores reflecting greater credibility, fact-checking likelihood, confidence, difficulty trusting online information, or trust. *p*-values are from Wilcoxon rank-sum or Kruskal–Wallis tests.

## Data Availability

The data presented in this study are available on request from the corresponding author. The data are not publicly available due to ethical restrictions.

## Supplementary Materials

The following supporting information can be downloaded at: https://www.mdpi.com/article/10.3390/pharmacy14030087/s1, Supplementary File S1. Flu Vaccine Study—IRAP; Supplementary File S2. SAS Analysis Code.

## Appendix A

**Table A1:** Economic burden by vaccination status among respondents with influenza-like illness.

Outcome	Not Vaccinated or Not Sure Median (IQR)	Vaccinated Median (IQR)	*p*-Value
Total direct out-of-pocket cost, dollars	48 (0–60)	16 (0–150)	0.498
Missed paid work, hours	16 (8–32)	11 (0–24)	0.264
Missed unpaid work or caregiving, hours	10.5 (0–16)	0 (0–10)	0.193
Missed work or school, days	2 (1–4)	2 (0–5)	0.817
Reduced usual activities, days	3 (2–10)	3 (0–5)	0.234

Note: *p*-values are from Wilcoxon rank-sum or Kruskal–Wallis tests.

## References

[1] Centers for Disease Control and Prevention (CDC) About Estimated Flu Burden. Updated 25 February 2026. https://www.cdc.gov/flu-burden/php/about/index.html.

[2] Centers for Disease Control and Prevention (CDC) (2026). 2024–2025 Influenza Season Summary: Severity, Disease Burden, and Burden Prevented. Flu Burden. https://www.cdc.gov/flu-burden/php/data-vis-vac/2024-2025-prevented.html.

[3] Centers for Disease Control and Prevention (CDC) Weekly US Influenza Surveillance Report: Key Updates for Week 13, ending 4 April 2026. FluView. https://www.cdc.gov/fluview/surveillance/2026-week-13.html.

[4] Grohskopf L.A., Blanton L.H., Ferdinands J.M., Reed C., Dugan V.G., Daskalakis D.C. (2025). Prevention and Control of Seasonal Influenza with Vaccines: Recommendations of the Advisory Committee on Immunization Practices—United States, 2025–26 Influenza Season. MMWR Morb. Mortal. Wkly. Rep..

[5] Centers for Disease Control and Prevention (CDC) (2026). Flu Vaccination Coverage, United States, 2024–25 Influenza Season. FluVaxView. https://www.cdc.gov/fluvaxview/coverage-by-season/2024-2025.html.

[6] Maloney P., Reeves E.L., Wielgosz K., Price A.M., Natarajan K., DeSilva M.B., Dascomb K., Klein N.P., Tartof S.Y., Irving S.A. (2026). Interim Estimates of 2025–26 Seasonal Influenza Vaccine Effectiveness—United States, September 2025–February 2026. MMWR Morb. Mortal. Wkly. Rep..

[7] Zhu S., Quint J., León T.M., Li N.J., Muldrew S., Porse C., Flannery B., Ellington S., Murray E.L., Sachdev D. (2026). Interim Estimates of 2025–26 Seasonal Influenza Vaccine Effectiveness—California, October 2025–January 2026. MMWR Morb. Mortal. Wkly. Rep..

[8] Molinari N.-A.M., Ortega-Sanchez I.R., Messonnier M.L., Thompson W.W., Wortley P.M., Weintraub E., Bridges C.B. (2007). The annual impact of seasonal influenza in the US: Measuring disease burden and costs. Vaccine.

[9] Putri W.C.W.S., Muscatello D.J., Stockwell M.S., Newall A.T. (2018). Economic burden of seasonal influenza in the United States. Vaccine.

[10] de Courville C., Cadarette S.M., Wissinger E., Alvarez F.P. (2022). The economic burden of influenza among adults aged 18 to 64: A systematic literature review. Influenza Other Respir. Viruses.

[11] Srivastav A., Lu P.-J., Amaya A., Dever J.A., Stanley M., Franks J.L., Scanlon P.J., Fisher A.M., Greby S.M., Nguyen K.H. (2023). Prevalence of influenza-specific vaccination hesitancy among adults in the United States, 2018. Vaccine.

[12] Hwang J. (2020). Health Information Sources and the Influenza Vaccination: The Mediating Roles of Perceived Vaccine Efficacy and Safety. J. Health Commun..

[13] Whitehead H.S., French C.E., Caldwell D.M., Letley L., Mounier-Jack S. (2023). A systematic review of communication interventions for countering vaccine misinformation. Vaccine.

[14] Kaplan E.L., Meier P. (1958). Nonparametric Estimation from Incomplete Observations. J. Am. Stat. Assoc..

[15] Cox D.R. (1972). Regression Models and Life Tables. R. Stat. Soc. J. Ser. B Methodol..

[16] Office of Disease Prevention and Health Promotion Increase the Proportion of People Who Get the Flu Vaccine Every Year—IID-09. Healthy People 2030|odphp.health.gov. https://odphp.health.gov/healthypeople/objectives-and-data/browse-objectives/vaccination/increase-proportion-people-who-get-flu-vaccine-every-year-iid-09.

[17] Bednarczyk R.A., Chu S.L., Sickler H., Shaw J., Nadeau J.A., McNutt L.A. (2015). Low uptake of influenza vaccine among university students: Evaluating predictors beyond cost and safety concerns. Vaccine.

[18] Walsh M.M., Parker A.M., Vardavas R., Nowak S.A., Kennedy D.P., Gidengil C.A. (2020). The Stability of Influenza Vaccination Behavior Over Time: A Longitudinal Analysis of Individuals Across 8 Years. Ann. Behav. Med..

[19] Schmid P., Rauber D., Betsch C., Lidolt G., Denker M.L. (2017). Barriers of Influenza Vaccination Intention and Behavior—A Systematic Review of Influenza Vaccine Hesitancy, 2005–2016. PLoS ONE.

[20] Lohmann S., Albarracín D. (2022). Trust in the public health system as a source of information on vaccination matters most when environments are supportive. Vaccine.

[21] Centers for Disease Control and Prevention (CDC) (2026). National and State-Specific Estimates of Settings Where Adults Received Influenza, Updated COVID-19, and RSV Vaccinations, 2023–2024 Respiratory Virus Season, United States. RespVaxView. https://www.cdc.gov/respvaxview/publications/national-state-vaccination-estimates.html.

[22] Burson R.C., Buttenheim A.M., Armstrong A., Feemster K.A. (2016). Community pharmacies as sites of adult vaccination: A systematic review. Hum. Vaccin. Immunother..

[23] Isenor J.E., Edwards N.T., Alia T.A., Slayter K.L., MacDougall D.M., McNeil S.A., Bowles S.K. (2016). Impact of pharmacists as immunizers on vaccination rates: A systematic review and meta-analysis. Vaccine.

[24] Basol M., Roozenbeek J., van der Linden S. (2020). Good news about bad news: Gamified inoculation boosts confidence and cognitive immunity against fake news. J. Cogn..

[25] Van der Linden S., Roozenbeek J., Compton J. (2020). Inoculating against fake news about COVID-19. Front. Psychol..

[26] Roozenbeek J., van der Linden S., Goldberg B., Rathje S., Lewandowsky S. (2022). Psychological inoculation improves resilience against misinformation on social media. Sci. Adv..

[27] Allen J., Howland B., Mobius M., Rothschild D., Watts D.J. (2020). Evaluating the fake news problem at the scale of the information ecosystem. Sci. Adv..

[28] Lewandowsky S., Ecker U.K.H., Cook J. (2017). Beyond Misinformation: Understanding and Coping with the “Post-Truth” Era. J. Appl. Res. Mem. Cogn..

[29] Bertolotti M., Catellani P. (2023). Counterfactual thinking as a prebunking strategy to contrast misinformation on COVID-19. J. Exp. Soc. Psychol..

