# Supplementary material for: Seasonal Influenza Vaccination Uptake, Illness and Economic Burden, and Vaccine Information Exposure Among Young Adults in the San Francisco Bay Area
Source: Pharmacy (Basel). 2026 Jun 18;14(3):87. doi: 10.3390/pharmacy14030087 (PMC13306451; doi:10.3390/pharmacy14030087)
Supplement: Supplementary file 1 [file pharmacy-14-00087-s001.zip › Flu Vaccine Study Supplementary Files/Supplementary File S2.pdf]

```
/*****************************************************************************************
 Title: Seasonal Influenza Vaccination Uptake, Illness and Economic Burden,
         and Vaccine Information Exposure Among Adults Aged 18 to 49 Years
         in the San Francisco Bay Area: A Cross-Sectional Study
 Purpose: Clean Qualtrics CSV export and run descriptive, vaccination uptake,
         time to recovery, and economic burden analyses.
 Authors:  Taiwo Opeyemi Aremu, Carinne Brody, Shadi Doroudgar, Ikenna Chidozie Ezejiaku, Shahin Teimourtash
 Affiliation: College of Pharmacy, Touro University California, Vallejo, CA, USA
*****************************************************************************************/

options nodate nonumber validvarname=any mprint mlogic symbolgen;

/* Import Qualtrics CSV datafile */
proc import datafile="/home/u58217951/FluVaccineStudy.csv"
    out=FluVac
    dbms=csv
    replace;
    guessingrows=max;
    getnames=yes;
run;

/* Formats */
proc format;
    value ynfmt       0='No' 1='Yes';
    value vaxfmt      0='Not vaccinated or not sure' 1='Vaccinated';
    value ilifmt      0='No or not sure' 1='Yes';
    value eventfmt    0='Censored, not fully recovered' 1='Recovered';
    value expfreqfmt  0='Never' 1='Rarely' 2='Sometimes' 3='Often' 4='Very often';
run;

/* Utility macro to extract first numeric value from a text field */
%macro firstnum(charvar, outvar);
    _tmp_value = strip(vvalue(&charvar));
    _tmp_upper = upcase(compbl(strip(_tmp_value)));

    if missing(_tmp_value) then &outvar = .;
    else if prxmatch('/^(NONE|N\/A|NA|NOT APPLICABLE|NO COST|NO|0)$/i', strip(_tmp_value)) then &outvar = 0;
    else if prxmatch('/[-+]?[0-9]+(\.[0-9]+)?/', _tmp_value) then
        &outvar = input(prxchange('s/.*?([-+]?[0-9]+(\.[0-9]+)?).*/$1/', 1, _tmp_value), ?? best32.);
    else &outvar = .;
%mend firstnum;

/* Clean and derive analysis variables */
data FluVac_clean;
    length
        age_group $12 county_live $30 county_work $30 primary_county $30
        gender_cat $30 race_grp $35 ethnicity $25 education_cat $45 income_cat $35
        insurance_cat $35 workstudy_setting $25 employment_status $200
        vax_month $20 vax_location $60 preferred_vax_site $80
        info_sources $500 easier_vax $500 acceptable_sites $500
        reason_novax $500 _tmp_value _tmp_upper $300;

    set FluVac;

    /* Remove Qualtrics metadata rows */
    if substrn(strip(ResponseId),1,2) ne 'R_' then delete;

    /* Administrative and eligibility variables */
    response_id = strip(ResponseId);
    progress_num = input(vvalue(Progress), ?? best32.);
    duration_sec = input(vvalue('Duration (in seconds)'n), ?? best32.);
    recaptcha_score = input(vvalue(Q_RecaptchaScore), ?? best32.);
    start_dt = input(vvalue(StartDate), ?? anydtdtm.);
    end_dt   = input(vvalue(EndDate), ?? anydtdtm.);
    recorded_dt = input(vvalue(RecordedDate), ?? anydtdtm.);
    survey_date = datepart(end_dt);
    format start_dt end_dt recorded_dt datetime20. survey_date date9.;

    complete_flag = (progress_num >= 90);
    region_eligible = (strip(QID24) = 'Yes');
    age_eligible = (strip(QID6) in ('18-24','25-34','35-44','45-49'));
    consent_flag = (strip(QID73) = 'I agree to participate');

    /* Core demographic variables */
    age_group = strip(QID6);
    %firstnum(Q59, age_exact);
```

```
if missing(age_exact) then do;
    if age_group='18-24' then age_midpoint=21.5;
    else if age_group='25-34' then age_midpoint=29.5;
    else if age_group='35-44' then age_midpoint=39.5;
    else if age_group='45-49' then age_midpoint=47;
end;
else age_midpoint = age_exact;

county_live = strip(QID8);
county_work = strip(QID10);
primary_county = county_live;
gender_cat = strip(Q60);
ethnicity = strip(Q61);
education_cat = strip(Q63);
income_cat = strip(Q64);
insurance_cat = strip(QID16);
workstudy_setting = strip(QID14);
employment_status = strip(QID13);

student_flag = (index(upcase(QID13),'STUDENT')>0);
employed_flag = (index(upcase(QID13),'EMPLOYED')>0 or index(upcase(QID13),'GIG')>0);

/* Race indicators and grouped race variable */
race_white = (index(upcase(Q62),'WHITE')>0);
race_black = (index(upcase(Q62),'BLACK')>0);
race_asian = (index(upcase(Q62),'ASIAN')>0 and index(upcase(Q62),'ALASKA')=0);
race_native = (index(upcase(Q62),'AMERICAN INDIAN')>0 or index(upcase(Q62),'ALASKA NATIVE')>0);
race_pacific = (index(upcase(Q62),'PACIFIC ISLANDER')>0 or index(upcase(Q62),'NATIVE HAWAIIAN')>0);
race_another = (index(upcase(Q62),'ANOTHER RACE')>0);
race_pnts = sum(of race_white race_black race_asian race_native race_pacific race_another);

if missing(Q62) then race_grp='Missing';
else if index(upcase(Q62),'PREFER NOT')>0 then race_grp='Prefer not to say';
else if race_pnts > 1 then race_grp='Multiple races';
else if race_white then race_grp='White';
else if race_black then race_grp='Black or African American';
else if race_asian then race_grp='Asian';
else if race_native or race_pacific or race_another then race_grp='Other race';
else race_grp='Other or missing';

/* Health conditions */
asthma_lung = (index(upcase(QID15),'ASTHMA')>0 or index(upcase(QID15),'LUNG')>0);
diabetes = (index(upcase(QID15),'DIABETES')>0);
heart_kidney = (index(upcase(QID15),'HEART')>0 or index(upcase(QID15),'KIDNEY')>0);
immunocomp = (index(upcase(QID15),'WEAKENED IMMUNE')>0);
pregnancy_recent = (index(upcase(QID15),'PREGNANCY')>0);
chronic_any = (asthma_lung or diabetes or heart_kidney or immunocomp or pregnancy_recent);

/* Primary vaccination outcome */
if strip(QID17)='Yes' then vax=1;
else if strip(QID17) in ('No','Not sure') then vax=0;
else vax=. ;

if strip(QID17)='Yes' then vax3='Yes';
else if strip(QID17)='No' then vax3='No';
else if strip(QID17)='Not sure' then vax3='Not sure';
else vax3='Missing';

vax_month = strip(QID18);
vax_location = strip(QID19);
reason_novax = strip(QID21);

if strip(QID22)='Yes' then prior_vax=1;
else if strip(QID22) in ('No','Not sure') then prior_vax=0;
else prior_vax=. ;

/* Influenza-like illness */
if strip(QID23)='Yes' then ili=1;
else if strip(QID23) in ('No','Not sure') then ili=0;
else ili=. ;

if strip(QID25)='One' then ili_episodes=1;
else if strip(QID25)='Two' then ili_episodes=2;
else if strip(QID25)='Three or more' then ili_episodes=3;
else ili_episodes=. ;

flu_test_positive = (strip(QID27)='Yes, positive');
```

```
flu_test_any = (index(upcase(QID27),'YES')>0);
covid_test_any = (index(upcase(QID28),'YES')>0);
care_seek_any = (index(upcase(Q24),'YES')>0);
antiviral_rx = (strip(QID30)='Yes');
hospitalized = (strip(QID31)='Yes');

/* Dates for symptom onset and recovery */
symptom_date = input(strip(QID26_1_TEXT), ?? anydtdte.);
if missing(symptom_date) then symptom_date = input(strip(QID26_2_TEXT), ?? anydtdte.);
recovery_date = input(strip(QID32_1_TEXT), ?? anydtdte.);
format symptom_date recovery_date date9.;

season_start = '01AUG2025'd;
season_end   = '30APR2026'd;
if not missing(symptom_date) and (symptom_date < season_start or symptom_date > season_end) then symptom_date = .;
if not missing(recovery_date) and (recovery_date < season_start or recovery_date > '30JUN2026'd) then recovery_date = .;

%firstnum(QID84_1_TEXT, recovery_days_num);
if recovery_days_num < 0 or recovery_days_num > 180 then recovery_days_num = .;

if not missing(symptom_date) and not missing(recovery_date) then recovery_days_date = recovery_date - symptom_date;
if recovery_days_date < 0 or recovery_days_date > 180 then recovery_days_date = .;

recovery_days = coalesce(recovery_days_num, recovery_days_date);
not_recovered = (strip(QID84)='I have not yet fully recovered' or strip(QID32)='I have not yet fully recovered');
recovery_not_sure = (strip(QID84)='Not sure' or strip(QID32)='Not sure');

/* Time-to-recovery endpoint: event = recovered to usual activities */
time_recovery = .;
event_recovered = .;
if ili=1 then do;
    if not missing(recovery_days) then do;
        time_recovery = recovery_days;
        event_recovered = 1;
    end;
    else if not_recovered=1 then do;
        event_recovered = 0;
        if not missing(symptom_date) and not missing(survey_date) then time_recovery = survey_date - symptom_date;
    end;
end;
if time_recovery < 0 or time_recovery > 180 then do;
    time_recovery = .;
    event_recovered = .;
end;

/* Functional burden */
%firstnum(Q82_1_TEXT, miss_workschool_days);
if strip(Q82)='Not applicable (not working or studying)' then miss_workschool_na=1;
else miss_workschool_na=0;
if miss_workschool_days < 0 or miss_workschool_days > 180 then miss_workschool_days = .;

%firstnum(Q83_1_TEXT, reduced_activity_days);
if reduced_activity_days < 0 or reduced_activity_days > 180 then reduced_activity_days = .;

any_care_help = .;
if strip(Q84)='No' then any_care_help=0;
else if not missing(Q84) and strip(Q84) ne 'Not sure' then any_care_help=1;

%firstnum(QID88_1_TEXT, care_help_hours);
if care_help_hours < 0 or care_help_hours > 1000 then care_help_hours = .;

/* Economic burden: direct costs and indirect hours */
%firstnum(Q87, oop_visit_cost);
%firstnum(Q88, oop_med_cost);
%firstnum(Q89, transport_delivery_cost);
%firstnum(Q90, other_oop_cost);

array costs[4] oop_visit_cost oop_med_cost transport_delivery_cost other_oop_cost;
do _i=1 to dim(costs);
    if costs[_i] < 0 or costs[_i] > 10000 then costs[_i]=.;
end;
if n(of oop_visit_cost oop_med_cost transport_delivery_cost other_oop_cost) > 0 then
    total_direct_cost = sum(of oop_visit_cost oop_med_cost transport_delivery_cost other_oop_cost);
else total_direct_cost = .;

%firstnum(Q93_1_TEXT, paid_work_hours_missed);
if paid_work_hours_missed < 0 or paid_work_hours_missed > 1000 then paid_work_hours_missed = .;
```

```
%firstnum(Q94_1_TEXT, unpaid_work_hours_missed);
if unpaid_work_hours_missed < 0 or unpaid_work_hours_missed > 1000 then unpaid_work_hours_missed = .;

paid_sick_leave = strip(Q95);

/* Vaccine information environment */
info_sources = strip(Q96);
preferred_vax_site = strip(Q97);
acceptable_sites = strip(Q76);
easier_vax = strip(Q98);

select (strip(Q99));
    when ('Never') neg_claim_freq=0;
    when ('Rarely') neg_claim_freq=1;
    when ('Sometimes') neg_claim_freq=2;
    when ('Often') neg_claim_freq=3;
    when ('Very often') neg_claim_freq=4;
    otherwise neg_claim_freq=. ;
end;
neg_claim_exposed = (neg_claim_freq > 0);
if missing(neg_claim_freq) then neg_claim_exposed=. ;

if strip(Q100)='Yes' then conflicting_info=1;
else if strip(Q100)='No' then conflicting_info=0;
else conflicting_info=. ;

%firstnum(Q103, neg_claim_cred_raw);
if index(Q103,'99')>0 then neg_claim_cred_raw=.;

%firstnum(Q104, factcheck_raw);
if index(Q104,'99')>0 then factcheck_raw=.;
if 1 <= factcheck_raw <= 5 then factcheck_score = 6 - factcheck_raw; /* higher = more likely to fact check */

%firstnum(Q105, confidence_accuracy);
if index(Q105,'99')>0 then confidence_accuracy=.;

%firstnum(Q106, hard_trust_online_raw);
if 1 <= hard_trust_online_raw <= 5 then hard_trust_online = 6 - hard_trust_online_raw; /* higher = harder to trust online

%firstnum(Q67, trust_healthprof);
%firstnum(Q69, trust_publichealth);
%firstnum(Q70, trust_influencers);
%firstnum(Q71, trust_familyfriends);
%firstnum(Q72, trust_newsmedia);

/* Scenario and opinion-change indicators */
scenario_lookup = (index(upcase(Q109),'LOOK UP')>0);
scenario_believe = (index(upcase(Q109),'BELIEVE')>0);
scenario_share = (index(upcase(Q109),'SHARE')>0);
scenario_ignore = (index(upcase(Q109),'IGNORE')>0);

if index(upcase(Q110),'YES')>0 then opinion_changed_online=1;
else if strip(Q110)='No' then opinion_changed_online=0;
else opinion_changed_online=. ;

/* Final analytic sample flag */
analytic_flag = (complete_flag=1 and region_eligible=1 and age_eligible=1 and consent_flag=1 and not missing(vax));

label
    vax = 'Influenza vaccination uptake this season'
    prior_vax = 'Influenza vaccination uptake last season'
    ili = 'Influenza-like illness this season'
    time_recovery = 'Days to recovery or censoring time'
    event_recovered = 'Recovery event indicator'
    total_direct_cost = 'Total direct out-of-pocket cost'
    neg_claim_freq = 'Frequency of exposure to negative flu vaccine claims'
    neg_claim_cred_raw = 'Credibility of negative flu vaccine claims, 1 to 5'
    factcheck_score = 'Fact-checking likelihood, higher means more likely'
    confidence_accuracy = 'Confidence identifying accurate flu vaccine information'
    hard_trust_online = 'Difficulty knowing which online health information to trust'
    trust_healthprof = 'Trust in health professional as flu vaccine information source'
    trust_publichealth = 'Trust in CDC or public health agencies'
    trust_influencers = 'Trust in social media influencers'
    trust_familyfriends = 'Trust in family and friends'
    trust_newsmedia = 'Trust in traditional news media';
```

```
    format vax vaxfmt. prior_vax chronic_any student_flag employed_flag region_eligible age_eligible
           consent_flag complete_flag ynfmt. ili ilifmt. event_recovered eventfmt.
           neg_claim_freq expfreqfmt.;

    drop _tmp_value _tmp_upper _i season_start season_end;
run;

/* Analysis dataset  */
data work.analysis;
    set FluVac_clean;
    where analytic_flag=1;
run;

/* Sample disposition */
proc sql;
    create table work.sample_disposition as
    select
        count(*) as n_raw_responses,
        sum(complete_flag) as n_progress_ge_90,
        sum(region_eligible) as n_region_eligible,
        sum(age_eligible) as n_age_eligible,
        sum(consent_flag) as n_consented,
        sum(analytic_flag) as n_primary_analysis,
        sum(ili=1 and analytic_flag=1) as n_ili_in_analysis,
        sum(analytic_flag=1 and not missing(time_recovery) and not missing(event_recovered)) as n_time_recovery_analysis
    from work.flu_clean;
quit;

/* ODS output file */
ods excel file="&outdir/flu_irap_sas_results.xlsx" style=journal
    options(sheet_interval='proc' embedded_titles='yes' frozen_headers='yes');

ods excel options(sheet_name='Sample disposition');
title 'Sample Disposition';
proc print data=work.sample_disposition noobs; run;

ods excel options(sheet_name='Participant characteristics');
title 'Participant Characteristics';
proc freq data=work.analysis;
    tables age_group gender_cat race_grp ethnicity county_live county_work insurance_cat
           education_cat income_cat student_flag employed_flag workstudy_setting chronic_any
           / missing nocum;
run;

ods excel options(sheet_name='Vaccination uptake');
title 'Influenza Vaccination Uptake';
proc freq data=work.analysis;
    tables vax vax3 QID17 prior_vax vax_month vax_location preferred_vax_site acceptable_sites easier_vax
           / missing nocum;
run;

title 'Vaccination Uptake with 95 Percent Confidence Interval';
proc freq data=work.analysis;
    tables vax / binomial(level='Vaccinated');
run;

ods excel options(sheet_name='Bivariable uptake');
title 'Bivariable Associations with Vaccination Uptake';
proc freq data=work.analysis;
    tables (age_group gender_cat race_grp ethnicity insurance_cat chronic_any prior_vax
            student_flag employed_flag workstudy_setting neg_claim_freq conflicting_info)
           * vax / chisq expected norow nocol nopercent;
run;

ods excel options(sheet_name='Uptake logistic model');
title 'Parsimonious Logistic Regression for Influenza Vaccination Uptake';
proc logistic data=work.analysis descending;
    class age_group(ref='18-24') gender_cat(ref='Man') race_grp(ref='White')
          insurance_cat(ref='Private insurance') / param=ref;
    model vax(event='Vaccinated') = age_group gender_cat race_grp insurance_cat chronic_any prior_vax
                                 neg_claim_freq neg_claim_cred_raw factcheck_score confidence_accuracy
                                 trust_healthprof trust_publichealth;
    oddsratio age_group;
    oddsratio gender_cat;
    oddsratio race_grp;
    oddsratio insurance_cat;
run;
```

```
/* Sensitivity analysis excluding Not sure vaccination responses */
ods excel options(sheet_name='Sensitivity vax yes no');
title 'Sensitivity Analysis: Vaccinated versus No, Excluding Not Sure';
proc logistic data=work.analysis(where=(strip(QID17) in ('Yes','No'))) descending;
    class age_group(ref='18-24') gender_cat(ref='Man') race_grp(ref='White')
          insurance_cat(ref='Private insurance') / param=ref;
    model vax(event='Vaccinated') = age_group gender_cat race_grp insurance_cat chronic_any prior_vax
                           neg_claim_freq neg_claim_cred_raw factcheck_score confidence_accuracy
                           trust_healthprof trust_publichealth;
run;

ods excel options(sheet_name='ILI burden');
title 'Influenza-like Illness Burden';
proc freq data=work.analysis;
    tables ili ili_episodes flu_test_any flu_test_positive covid_test_any care_seek_any antiviral_rx
           hospitalized any_care_help paid_sick_leave / missing nocum;
run;

title 'Functional Burden Among Respondents Reporting Influenza-like Illness';
proc means data=work.analysis(where=(ili=1)) n nmiss mean std median q1 q3 min max maxdec=2;
    var recovery_days time_recovery miss_workschool_days reduced_activity_days care_help_hours
        paid_work_hours_missed unpaid_work_hours_missed;
run;

ods excel options(sheet_name='Time to recovery');
title 'Time to Recovery Among Respondents Reporting Influenza-like Illness';
proc means data=work.analysis(where=(ili=1 and not missing(time_recovery))) n nmiss mean std median q1 q3 min max maxdec=2;
    var time_recovery;
run;

proc freq data=work.analysis(where=(ili=1));
    tables event_recovered*QID17 / missing chisq;
run;

/* Kaplan-Meier time-to-recovery analysis. Event=1 means recovered. HR>1 in PHREG means faster recovery. */
title 'Kaplan-Meier Time to Recovery by Vaccination Status';
proc lifetest data=work.analysis(where=(ili=1 and not missing(time_recovery) and not missing(event_recovered)))
              plots=survival(atrisk);
    time time_recovery*event_recovered(0);
    strata vax / test=logrank;
run;

title 'Exploratory Cox Model for Time to Recovery';
proc phreg data=work.analysis(where=(ili=1 and not missing(time_recovery) and not missing(event_recovered)));
    class age_group(ref='18-24') gender_cat(ref='Man') / param=ref;
    model time_recovery*event_recovered(0) = vax age_group gender_cat chronic_any care_seek_any / ties=efron;
    hazardratio vax;
run;

ods excel options(sheet_name='Economic burden');
title 'Direct and Indirect Economic Burden Among Respondents Reporting Influenza-like Illness';
proc means data=work.analysis(where=(ili=1)) n nmiss mean std median q1 q3 min max maxdec=2;
    var oop_visit_cost oop_med_cost transport_delivery_cost other_oop_cost total_direct_cost
        paid_work_hours_missed unpaid_work_hours_missed miss_workschool_days reduced_activity_days;
run;

title 'Economic Burden by Vaccination Status Among Respondents Reporting Influenza-like Illness';
proc means data=work.analysis(where=(ili=1)) n mean std median q1 q3 maxdec=2;
    class vax;
    var total_direct_cost paid_work_hours_missed unpaid_work_hours_missed miss_workschool_days reduced_activity_days;
run;

proc npar1way data=work.analysis(where=(ili=1)) wilcoxon;
    class vax;
    var total_direct_cost paid_work_hours_missed unpaid_work_hours_missed miss_workschool_days reduced_activity_days;
run;

ods excel options(sheet_name='Information environment');
title 'Vaccine Information Exposure, Credibility, Trust, and Fact Checking';
proc freq data=work.analysis;
    tables neg_claim_freq neg_claim_exposed conflicting_info Q101 Q65 Q66 scenario_lookup
           scenario_believe scenario_share scenario_ignore opinion_changed_online / missing nocum;
run;

proc means data=work.analysis n nmiss mean std median q1 q3 min max maxdec=2;
    var neg_claim_cred_raw factcheck_score confidence_accuracy hard_trust_online
```

```
        trust_healthprof trust_publichealth trust_influencers trust_familyfriends trust_newsmedia;
run;

ods excel options(sheet_name='Information by uptake');
title 'Vaccine Information Variables by Influenza Vaccination Uptake';
proc means data=work.analysis n mean std median q1 q3 maxdec=2;
    class vax;
    var neg_claim_cred_raw factcheck_score confidence_accuracy hard_trust_online
        trust_healthprof trust_publichealth trust_influencers trust_familyfriends trust_newsmedia;
run;

proc npar1way data=work.analysis wilcoxon;
    class vax;
    var neg_claim_cred_raw factcheck_score confidence_accuracy hard_trust_online
        trust_healthprof trust_publichealth trust_influencers trust_familyfriends trust_newsmedia;
run;

ods excel options(sheet_name='Data quality checks');
title 'Data Quality Checks and Potential Outliers';
proc freq data=work.flu_clean;
    tables complete_flag region_eligible age_eligible consent_flag analytic_flag
           Q_RecaptchaStatus Q_DuplicateRespondentStatus Q_BallotBoxStuffing / missing nocum;
run;

proc means data=work.flu_clean n nmiss mean std median min max maxdec=2;
    var progress_num duration_sec recaptcha_score age_exact recovery_days time_recovery
        total_direct_cost paid_work_hours_missed unpaid_work_hours_missed;
run;

ods excel close;

title;
footnote;
```
