# Supplementary material for: Seasonal Influenza Vaccination Uptake, Illness and Economic Burden, and Vaccine Information Exposure Among Young Adults in the San Francisco Bay Area
Source: Pharmacy (Basel). 2026 Jun 18;14(3):87. doi: 10.3390/pharmacy14030087 (PMC13306451; doi:10.3390/pharmacy14030087)
Supplement: Supplementary file 1 [file pharmacy-14-00087-s001.zip › Flu Vaccine Study Supplementary Files/Supplementary File S1.pdf]

Flu Vaccine Study - IRAP

Start of Block: Survey Consent

Title of the study: Uptake, Health Impact, and Economic Implications of Seasonal Influenza Among Young Adults in the San Francisco Bay Area **The purpose of this study:** Touro University California College of Pharmacy is conducting a brief online survey to learn about young adults' flu vaccination, any flu-like illness this season, recovery time, and related costs. Results will help inform local public health messaging and resources for young adults in the San Francisco Bay Area. . **What you will do for this study:** If you agree to participate, you will complete an online Qualtrics survey about your vaccination history, any flu-like illness this season, recovery time, health beliefs, and basic demographics. The survey is anonymous. **Time required for participation:** The survey will take about 10-12 minutes to complete. **Possible risks from your participation:** You may feel minor discomfort answering personal health questions. There is a very small risk of loss of confidentiality, which we will minimize through the protections described below. **Benefits from your participation:** Your input will provide valuable information to help improve local flu vaccination outreach and understanding of illness and recovery among young adults. There is no direct clinical benefit to you. **Incentives** After finishing the survey, you may enter an optional drawing for one of 50 electronic gift cards valued at $20 each. Entry information is collected on a separate form that is not linked to your survey responses. Odds of winning depend on the number of entries. One entry per completed survey. Winners will receive their prize electronically. **Confidentiality** The survey does not ask for your name or other direct identifiers, and IP address storage will be disabled. De-identified survey data will be stored in a secure University Box folder accessible only to CITI-trained study personnel listed on the IRB. Drawing contact information will be collected and stored separately, used only to deliver prizes, and deleted within 60 days after prize distribution. Results will be reported in aggregate. **Right to Withdraw** Participation is completely voluntary. You may skip any question or stop the survey at any time without penalty. **IRB Approval and Contact** This study has been reviewed by Touro University California's Institutional Review Board (IRB). If you have questions about the study, please contact the Principal Investigator, Dr. Aremu at taremu@touro.edu If you have questions about your rights as a research participant, contact the IRB Chair at (707) 638-5236 or twong@touro.edu

Page Break

Eligibility: Do you live, work, or study in any of these Bay Area counties: Alameda, Contra Costa, Marin, Napa, San Francisco, San Mateo, Santa Clara, Solano, Sonoma?

- ◯ Yes (1)
- ◯ No (2)

Skip To: End of Survey If Do you live in these area?(Alameda, Contra Costa, Marin, Napa, San Fracisco = No

Page Break

What is your age?

- ◯ less than 18 (7)
- ◯ 18-24 (2)
- ◯ 25-34 (3)
- ◯ 35-44 (4)
- ◯ 45-49 (5)
- ◯ 50 or older (6)

Skip To: End of Survey If What is your age? = 50 or older

Skip To: End of Survey If What is your age? = less than 18

Statement of Consent: By selecting “I agree” below, you confirm that you are 18-49 years old, live, work, or study in the San Francisco Bay Area, have read the information above, and voluntarily agree to participate in this anonymous survey.

- ◯ I agree to participate (1)
- ◯ I do not agree to participate (2)

Skip To: End of Survey If Statement of Consent = I do not agree to participate

End of Block: Survey Consent

Start of Block: Season definition

Season definition: *For this survey, “this influenza season” means August 1, 2025 through April 30, 2026.*

End of Block: Season definition

Start of Block: Section 1

Where do you live most of the time? (Bay Area County)

- ○ Alameda (1)
- ○ Contra Costa (2)
- ○ Marin (3)
- ○ Napa (4)
- ○ San Francisco (5)
- ○ San Mateo (6)
- ○ Santa Clara (7)
- ○ Solano (8)
- ○ Sonoma (9)
- ○ Outside the Bay Area (10)
- ○ Prefer not to say (11)

Page Break

Where do you work or study most of the time? (Mon-Fri, typical week)

- Alameda (1)
- Contra Costa (2)
- Marin (3)
- Napa (4)
- San Francisco (5)
- San Mateo (6)
- Santa Clara (7)
- Solano (8)
- Sonoma (9)
- Multiple counties about equally (10)
- Remote only / no regular location (11)
- Not applicable (not working or studying) (12)
- Not Applicable (13)
- Prefer not to say (14)

Page Break

What is your home zip code?

____

Page Break

Which applies to you right now? **(Check all that apply).**

- ☐ Full-time student (1)
- ☐ Part-time student (2)
- ☐ Employed full time (3)
- ☐ Employed part time (4)
- ☐ Not working for pay (5)
- ☐ Gig or shift worker (6)
- ☐ Prefer not to say (7)

Page Break

What best describes your usual work or study setting?

- ○ In person (1)
- ○ Hybrid (2)
- ○ Remote (3)
- ○ Not applicable (4)

End of Block: Section 1

Start of Block: Section 2. Health and access

Do you have any of the following that a clinician has told you about? **(Check all that apply).**

- ▢ Asthma or chronic lung disease (1)
- ▢ Diabetes (2)
- ▢ Heart or kidney disease (3)
- ▢ Weakened immune system (4)
- ▢ Pregnancy in the most recent season (5)
- ▢ None of the above (6)
- ▢ Prefer not to say (7)

What kind of health coverage do you have?

- ◯ Private insurance (1)
- ◯ Medi-Cal (2)
- ◯ Medicare (3)
- ◯ Student health plan (4)
- ◯ Uninsured (5)
- ◯ Prefer not to say (6)

End of Block: Section 2. Health and access

Start of Block: Section 3. Influenza vaccination uptake (November-ready)

Note for participants: The questions below refer to the 2025 to 2026 influenza season, from August 1, 2025 through April 30, 2026.

Page Break

Have you received an influenza vaccine this season?

- Yes (1)
- No (2)
- Not sure (3)

Skip To: QID20 If Have you received an influenza vaccine this season? = No

Skip To: QID20 If Have you received an influenza vaccine this season? = Not sure

In which month did you receive your influenza vaccine this season?

- August 2025 (1)
- September 2025 (2)
- October 2025 (3)
- November 2025 (4)
- December 2025 (5)
- January 2026 (6)
- Not sure (7)

Where did you receive it?

- ○ Doctor office or clinic (1)
- ○ Pharmacy (2)
- ○ Workplace or campus event (3)
- ○ Community site (4)
- ○ Other (5) ____

Page Break

Display this question:

If Have you received an influenza vaccine this season? = No

Or Have you received an influenza vaccine this season? = Not sure

How likely are you to get the influenza vaccine this season?

- ◯ Very likely (1)
- ◯ Somewhat likely (2)
- ◯ Not sure (3)
- ◯ Somewhat unlikely (4)
- ◯ Very unlikely (5)

Display this question:

If Have you received an influenza vaccine this season? = No

Or Have you received an influenza vaccine this season? = Not sure

What were the main reasons for not getting the influenza vaccine this season? Select up to three

- ☐ Concern about side effects (1)
- ☐ Do not think I need it (2)
- ☐ Cost or insurance (3)
- ☐ Time or scheduling barriers (4)
- ☐ Not sure where to go (5)
- ☐ Past negative experience (6)
- ☐ Prefer natural immunity (7)
- ☐ Other (specify) (8) ____________________

Did you receive an influenza vaccine last season?

- ○ Yes (1)
- ○ No (2)
- ○ Not sure (3)

End of Block: Section 3. Influenza vaccination uptake (November-ready)

Start of Block: Section 4-9

The next questions ask about flu-like illness during the 2025 to 2026 season defined as August 1, 2025 through April 30, 2026. *Flu-like illness means fever (≥100.4°F / 38°C or felt feverish) with cough or sore throat. We know these symptoms can also occur with COVID-19. Please answer based on your symptoms; we will also ask about any flu/COVID testing.*

In the current flu season, have you had an illness that met the flu definition above?

- ◯ Yes (1)
- ◯ No (2)
- ◯ Not sure (3)

Skip To: Q96 If In the current flu season, have you had an illness that met the flu definition above? = No

Skip To: Q96 If In the current flu season, have you had an illness that met the flu definition above? = Not sure

Page Break

Since August 1, 2025, how many separate flu-like illness episodes have you had?

- One (1)
- Two (2)
- Three or more (3)
- Not sure (4)

Page Break

Thinking about your **most recent** episode, on what date did symptoms begin?

- ○ Date (Month, day, year) (1) ____
- ○ Not sure (enter month & year or approximate date) (2) ____

During that episode, did you take a test for flu/influenza?

- ○ Yes, positive (2)
- ○ Yes, negative (4)
- ○ Yes, result pending or unknown (5)
- ○ No test (6)

Page Break

During that episode, did you test for COVID-19?

- ○ Yes, Positive (1)
- ○ Yes, Negative (4)
- ○ Yes, result pending or unknown (5)
- ○ No test (6)

Q24 Did you see a clinician or seek care for that episode?

- ○ Yes, in person (1)
- ○ Yes, telehealth (4)
- ○ No (5)

Page Break

Were you prescribed antiviral medication (for example, oseltamivir/Tamiflu, baloxavir/Xofluza)?

- ○ Yes (1)
- ○ No (4)
- ○ Not sure (5)

Were you admitted to a hospital overnight for that episode?

- ○ Yes (1)
- ○ No (7)

Skip To: QID84 If Were you admitted to a hospital overnight for that episode? = No

---

Page Break

Display this question:

If Were you admitted to a hospital overnight for that episode? = Yes

On what date did you fully recover to your usual activities?

- ◯ Date (MM/DD/YYYY) (1) ____
- ◯ I have not yet fully recovered (4)
- ◯ Not sure (5)

Page Break

Q80 *These questions refer to the most recent flu-like illness you reported above.*

Page Break

How many days passed from the start of symptoms until you felt recovered enough to return to your usual activities? *["Usual activities" = your normal work/school and daily routines. Enter 0 if you did not miss any time].*

- ○ Number of days (1) ____
- ○ I have not yet fully recovered (2)
- ○ Not sure (3)

Page Break

Q82 How many days of paid or unpaid work or school did you miss during that episode? *[Hint: Count full days you did not attend. Enter 0 if none].*

- ○ Number of days (1) ____
- ○ Not applicable (not working or studying) (2)
- ○ Not sure (3)

Page Break

Q83 On how many days did you reduce your normal activities by at least half? *[Hint: Count days you scaled back work/school/household tasks by ~50% or more].*

- ○ Number of days (1) ____

- ○ Not sure (2)

Page Break

Q84 Did anyone provide you with care or help because of this illness? **(Check all that apply).**

- ▢ No (1)
- ▢ Rides or arranging transportation (2)
- ▢ Meal prep or delivering food (3)
- ▢ Grocery or medication pickup (4)
- ▢ Childcare or pet care (5)
- ▢ Household chores (laundry, cleaning) (6)
- ▢ In-person care (checking vitals, helping with meds) (7)
- ▢ Other help (specify) (8) ____
- ▢ Not sure (9)

Page Break

Display this question:

If Did anyone provide you with care or help because of this illness? (Check all that apply). = Rides or arranging transportation

Or Did anyone provide you with care or help because of this illness? (Check all that apply). = Meal prep or delivering food

Or Did anyone provide you with care or help because of this illness? (Check all that apply). = Grocery or medication pickup

Or Did anyone provide you with care or help because of this illness? (Check all that apply). = Childcare or pet care

Or Did anyone provide you with care or help because of this illness? (Check all that apply). = Household chores (laundry, cleaning)

Or Did anyone provide you with care or help because of this illness? (Check all that apply). = In-person care (checking vitals, helping with meds)

Or Did anyone provide you with care or help because of this illness? (Check all that apply). = Other help (specify)

About how many total hours of care/help did they provide during this illness? *[Hint: Count both in-person and practical help done because you were sick. Do not include routine help you would have received anyway].*

○ Hours (1) ____

○ Not sure (2)

Page Break

Q86 ***If a cost did not occur, enter 0. Estimates are fine.***

Q87 Out of pocket costs for medical visits and copays

____

____

____

____

____

Page Break

Q73 ***If a cost did not occur, enter 0. Estimates are fine.***

Q88 Out of pocket costs for medications such as pain relievers or antivirals medications

____

____

____

____

____

Page Break

Q74 ***If a cost did not occur, enter 0. Estimates are fine.***

Q89 Transportation or delivery costs related to care or supplies

____

____

____

____

____

Page Break

Q75 ***If a cost did not occur, enter 0. Estimates are fine.***

Q90 Other out of pocket costs

____

____

____

____

____

Page Break

Q93 Hours of paid work missed

○ Hours (1) ____

○ Not applicable (2)

Page Break

Q94 Hours of unpaid work or caregiving missed

- Hours (1) ____
- Not applicable (2)

Page Break

Q95 Did you receive paid sick leave for days missed?

- ○ Yes, for all days (1)
- ○ Yes, for some days (2)
- ○ No (3)
- ○ Not applicable (4)

Page Break

Q96 Which information sources do you use for influenza or vaccine information? **Select up to three.**

- ☐ Health care provider (1)
- ☐ Pharmacist (2)
- ☐ Public health or government site (3)
- ☐ School or campus communication (4)
- ☐ Social media or influencers (5)
- ☐ Friends or family (6)
- ☐ News or radio (7)
- ☐ Employer (8)
- ☐ Other (specify) (9) ____

Page Break

Q97 Where would you prefer to receive a vaccine, if you choose to get one? **Select the one you prefer the most**

- ○ Doctor office or clinic (1)
- ○ Pharmacy (2)
- ○ Workplace or campus event (3)
- ○ Community site (4)
- ○ Place of worship/religious gathering (5)
- ○ I do not plan to get a vaccine (6)
- ○ Other (specify) (7) ____

Page Break

Q76 Which places would be acceptable for you to receive a vaccine? **(Check all that apply).**

- ☐ Doctor office or clinic (1)
- ☐ Pharmacy (2)
- ☐ Workplace or campus event (3)
- ☐ Community site (4)
- ☐ Place of worship/religious gathering (5)
- ☐ I do not plan to get a vaccine (6)
- ☐ Other (specify) (7) ____

Page Break

Q98 What would make it easier for you to get vaccinated? **Select up to three.**

- ▢ Lower or no cost (1)
- ▢ Evening or weekend hours (2)
- ▢ Walk in with no appointment (3)
- ▢ On site at work or campus (4)
- ▢ More information from a trusted source (5)
- ▢ Help with transportation (6)
- ▢ I do not plan to get vaccinated (7)
- ▢ Other (specify) (8) ____

Page Break

Q99 In the past 6 months, how often have you come across information online or on social media claiming that flu vaccines are unsafe or unnecessary?

- Never (1)
- Rarely (2)
- Sometimes (3)
- Often (4)
- Very often (5)

Q100 Have you ever seen or heard conflicting information about the flu vaccine from multiple sources?

- Yes (1)
- No (2)

Page Break

Q101 Where have you seen or heard claims or discussions questioning the flu vaccine? **(Check all that apply)**

- ☐ TikTok (1)
- ☐ Instagram (2)
- ☐ YouTube (3)
- ☐ X (Twitter) (4)
- ☐ Facebook (5)
- ☐ Friends/family (6)
- ☐ News media (7)
- ☐ Podcasts (8)
- ☐ Other (please specify) (9) ____

Page Break

Q65 Which of the following statements have you seen or heard before? **(Check all that apply)**

- ☐ The flu vaccine can give you the flu (1)
- ☐ The flu vaccine weakens your immune system (2)
- ☐ Healthy young adults don’t need a flu shot (3)
- ☐ The flu vaccine contains harmful chemicals or toxins (4)
- ☐ Flu vaccines are not effective because the flu virus keeps changing (5)
- ☐ None (6)

Page Break

Q66 Which of the following statements do you think are true? **(Check all that apply)**

- ☐ The flu vaccine can give you the flu (1)
- ☐ The flu vaccine weakens your immune system (2)
- ☐ Healthy young adults don’t need a flu shot (3)
- ☐ The flu vaccine contains harmful chemicals or toxins (4)
- ☐ Flu vaccines are not effective because the flu virus keeps changing (5)
- ☐ None (6)

Page Break

Q103 How credible did you find the negative claims about flu vaccines that you have seen or heard?

- ◯ 1 - Not credible at all (1)
- ◯ 2 - Slightly credible (2)
- ◯ 3 - Moderately credible (3)
- ◯ 4 - Very credible (4)
- ◯ 5 - Extremely credible (5)
- ◯ 99 - I have not seen or heard negative claims about flu vaccines (6)

Page Break

Q104 When you see or hear information that questions vaccine efficacy or safety, how likely are you to fact-check or look for official sources?

- 1 - Very Likely (1)
- 2 - Somewhat likely (2)
- 3 - Neither likely nor unlikely (3)
- 4 - Somewhat unlikely (4)
- 5 - Not at all likely (5)
- 99 - I have not seen or heard negative claims (6)

Page Break

Q105 How confident are you in your ability to tell whether information about flu vaccines is accurate or not?

- ○ 1 - Not at all confident (1)
- ○ 2 - Slightly confident (2)
- ○ 3 - Moderately confident (3)
- ○ 4 - Very confident (4)
- ○ 5 - Extremely confident (5)
- ○ 99 - Not sure (6)

Page Break

Q106 It’s hard for me to know which health information online I can trust (Agree/Disagree).

- ○ 1 - Strongly agree (1)
- ○ 2 - Agree (2)
- ○ 3 - Neither agree nor disagree (3)
- ○ 4 - Disagree (4)
- ○ 5 - Strongly disagree (5)

Page Break

Q67 How much do you trust your **health professional** as a source for information about flu vaccines? (1 = None at all → 5 = Completely)

- ◯ 1 - None at all (1)
- ◯ 2 - A little (2)
- ◯ 3 - Somewhat/a moderate amount (3)
- ◯ 4 - A lot (4)
- ◯ 5 - Completely (5)
- ◯ Not applicable, I do not have a health care professional (6)

Q69 How much do you trust the **CDC or other public health agencies** as a source for information about flu vaccines? (1 = None at all → 5 = Completely)

- ◯ 1 - None at all (1)
- ◯ 2 - A little (2)
- ◯ 3 - Somewhat/a moderate amount (3)
- ◯ 4 - A lot (4)
- ◯ 5 - Completely (5)
- ◯ Not sure (6)

Page Break

Q70 How much do you trust **social media influencers** as a source for information about flu vaccines? (1 = None at all → 5 = Completely)

- 1 - None at all (1)
- 2 - A little (2)
- 3 - Somewhat/a moderate amount (3)
- 4 - A lot (4)
- 5 - Completely (5)
- I do not follow social media influencers (6)

Q71 How much do you trust **family and friends** as a source for information about flu vaccines? (1 = None at all → 5 = Completely)

- 1 - None at all (1)
- 2 - A little (2)
- 3 - Somewhat/a moderate amount (3)
- 4 - A lot (4)
- 5 - Completely (5)
- Not sure (6)

Q72 How much do you trust **traditional news media** as a source for information about flu vaccines? (1 = None at all → 5 = Completely)

- 1 - None at all (1)
- 2 - A little (2)
- 3 - Somewhat/a moderate amount (3)
- 4 - A lot (4)
- 5 - Completely (5)
- Not sure (6)

Page Break

Q109 Imagine you see a viral social media post saying: "Flu shots don't work because the virus always mutates/change." What would you most likely do?

- ☐ Believe it — you know that the flu virus does change often (1)
- ☐ Look up information from a trusted medical/public health source (2)
- ☐ Share it to warn others (3)
- ☐ Ignore it — I don't know what's true (4)

Q110 Have you ever changed your opinion about the flu vaccine because of something you saw or heard online?

- ○ Yes (please describe) (1) ____
- ○ No (2)

End of Block: Section 4-9

Start of Block: Block 10

Q59 Age

____

____

____

____

____

Page Break

Q60 Gender

- Woman (4)
- Man (5)
- Nonbinary (6)
- Prefer to self-describe (7) ____
- Prefer not to say (8)

Q61 Hispanic or Latino origin:

- Yes (1)
- No (4)
- Prefer not to say (5)

Page Break

Q62 Race (Select all that apply)

- ☐ American Indian or Alaska Native (1)
- ☐ Asian (4)
- ☐ Black or African American (5)
- ☐ Native Hawaiian or Pacific Islander (6)
- ☐ White (7)
- ☐ Another race (8)
- ☐ Prefer not to say (9)

Page Break

Q63 Highest level of education

- ○ High school or less (1)
- ○ Some college (4)
- ○ Associate degree (5)
- ○ Bachelor's degree (6)
- ○ Graduate or professional degree (7)
- ○ Prefer not to say (8)

Page Break

Q64 Household income before taxes (in USD)

- ○ Less than 25,000 (1)
- ○ 25,000 to 49,999 (4)
- ○ 50,000 to 74,999 (5)
- ○ 75,000 to 99,999 (6)
- ○ 100,000 to 149,999 (7)
- ○ 150,000 or more (8)
- ○ Prefer not to say (9)

End of Block: Block 10

Start of Block: Block 11

Thank you for participating in the survey. This is the end of the survey! STRICTLY OPTIONAL: Fifty (50) participants will be randomly selected through a drawing to receive $20 e-gift card. If you wish to partake in the drawing, **please click on this LINK**, to provide your contact information (email or phone number) for your gift IF you win. Please NOTE that your contact Information is not linked to your survey responses.

End of Block: Block 11
